# Climate and Land Use Controls on Soil Organic Carbon in the Loess Plateau Region of China

**DOI:** 10.1371/journal.pone.0095548

**Published:** 2014-05-01

**Authors:** Yaai Dang, Wei Ren, Bo Tao, Guangsheng Chen, Chaoqun Lu, Jia Yang, Shufen Pan, Guodong Wang, Shiqing Li, Hanqin Tian

**Affiliations:** 1 State Key Laboratory of Soil Erosion and Dryland Farming on the Loess Plateau, Institute of Soil and Water Conservation, Northwest A&F University, Yangling, Shaanxi, China; 2 International Center for Climate and Global Change Research, School of Forestry & Wildlife Sciences, Auburn University, Auburn, Alabama, United States of America; 3 College of Science, Northwest A&F University, Yangling, Shaanxi, China; University of Maryland, United States of America

## Abstract

The Loess Plateau of China has the highest soil erosion rate in the world where billion tons of soil is annually washed into Yellow River. In recent decades this region has experienced significant climate change and policy-driven land conversion. However, it has not yet been well investigated how these changes in climate and land use have affected soil organic carbon (SOC) storage on the Loess Plateau. By using the Dynamic Land Ecosystem Model (DLEM), we quantified the effects of climate and land use on SOC storage on the Loess Plateau in the context of multiple environmental factors during the period of 1961–2005. Our results show that SOC storage increased by 0.27 Pg C on the Loess Plateau as a result of multiple environmental factors during the study period. About 55% (0.14 Pg C) of the SOC increase was caused by land conversion from cropland to grassland/forest owing to the government efforts to reduce soil erosion and improve the ecological conditions in the region. Historical climate change reduced SOC by 0.05 Pg C (approximately 19% of the total change) primarily due to a significant climate warming and a slight reduction in precipitation. Our results imply that the implementation of “Grain for Green” policy may effectively enhance regional soil carbon storage and hence starve off further soil erosion on the Loess Plateau.

## Introduction

Soil organic carbon (SOC), the major component of soil organic matter, plays a key role in the terrestrial carbon cycle and thus has drawn great attention from scientific community. It is a dynamic component of terrestrial systems, affecting carbon exchange between terrestrial ecosystem and the atmosphere [1], [2]. SOC storage is nearly three times as large as carbon storage in vegetation and twice as large as global atmospheric carbon storage [3]. Soil has higher potential to sequester more carbon (such as converting the type of land use) in the future [2], [4], therefore, increasing soil carbon storage is one of the most economical and effective ways to alleviate the greenhouse effect, which has become a hot scientific and political issue during the past decades.

Changes in climate and land use, caused by both natural and anthropogenic processes, have greatly influenced the terrestrial carbon balance during the past decades [5], [6], [7], [8]. It was reported that about one fourth of anthropogenic CO_2_ emissions were due to land cover and land use change (LCLUC), especially deforestation [9]. Long-term experimental studies have confirmed that SOC is highly sensitive to land conversion from natural ecosystems, such as forest or grassland, to agricultural land, resulting in substantial SOC loss [6], [10]. In addition, LCLUC may also cause carbon depletion by influencing soil respiration [11]. It was estimated that global carbon release from SOC mineralization owing to agricultural activities was approximately 0.80 Pg C/year (1 Pg = 10^15^ g) [12]. Globally, land use change resulted in a carbon release of (1.6±0.8) Pg C per year to the atmosphere during the period of 1990s [13]. However, the effects of conversions from cropland to grassland/forest on the SOC storage have not been fully understood and there still remains large uncertainty.

**Figure 1:**
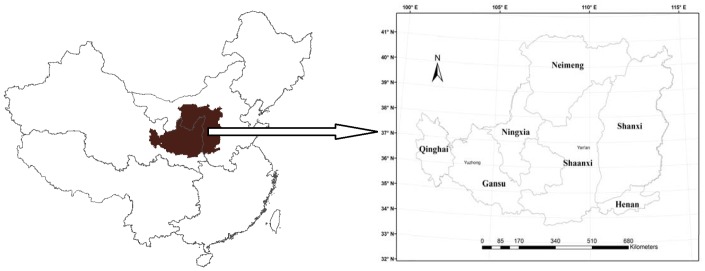
Location of the Loess Plateau, China.

The Loess Plateau of China (Figure 1), located in the geographic center of China (33^o^43′N 100^o^54′E to 41^o^16′N 114^o^33′E), covers a total area of 628,000 km^2^, which is about 6.5% of China’s total land area. The Loess Plateau is characterized by highly erodible soils, steep slopes, being subjected to heavy rain, and low vegetation coverage due to excess exploitation of land resource and improper land use [4], [14]. During the past decades, serious soil erosions caused by natural and anthropogenic disturbances (e.g., climate change, natural disasters, LCLUC etc.) occurred in the area of the Loess Plateau. Previous reports also indicated that the warming and drying climate in this region has significantly aggravated soil erosion [15]. As a result, the large amount of fine surface soil eroded from the loess area is transported into the Yellow River and acts as the main source of sediment of this river, which runs through the Loess Plateau and is considered to be the most turbid river in the world. Due to these disturbances, soil carbon storage on the Loess Plateau is much lower compared to other regions in China [16]. In general, adjusting the land use pattern so as to restore the degraded ecosystems and to modify the local rural income structure is regarded as the main measures to control soil and water erosion and to improve farmers’ living conditions on the Loess Plateau. Since the 1950s, a series of conservation policies have been implemented in this region, such as extensive tree planting since the 1970s, integrated soil erosion controls on the watershed scale in the 1980s and the 1990s [17], [18], and the government-funded project “Grain for Green” in 1999, aiming at transforming the low-yield slope cropland into grassland/forest. The implementation of these policies improved vegetation coverage, altered land use patterns, and changed the SOC storage. Although many field experiments have been performed to explore the impacts of both drying and warming climate and LCLUC on soil carbon storage on the Loess Plateau [19], [20], little attention has been paid to the regional impacts of these factors and their interactions.

Over recent decades, many field observations and control experiments have been conducted to explore the effects of climate and land use change on the SOC in this region and make it possible to study the regional effects of climate change and LCLUC on SOC. In addition, many approaches, including eddy covariance flux tower, inventory, remote sensing techniques, forward and inversion models, have been used to examine the regional carbon budget on the Loess Plateau [21], [22], [23], [24], [25]. Among them, process-based ecosystem modeling is one of the most effective approaches to estimate regional SOC storage and fluxes in different terrestrial ecosystems driven by multiple global changes factors [7], [11], [24], [26], [27]. To address the effects of changes in climate, land use, and other environmental factors on SOC storage in this region, the Dynamic Land Ecosystem Model (DLEM), a highly integrated process-based model [28], was applied to evaluate the spatial and temporal patterns of SOC storage on the Loess Plateau during 1961–2005. The objectives of this study are: 1) to investigate the temporal and spatial patterns of SOC storage on the Loess Plateau; and 2) to identify the relative contribution of climate and land use changes to the SOC storage changes.

## Methods

### Model Description

The DLEM is a process-based terrestrial ecosystem model, which aims at simulating the impacts of natural and anthropogenic disturbances on the structure and functions of terrestrial ecosystems over the spatial and temporal contexts. The DLEM has been widely used to simulate the effects of climate variability and change, elevated atmospheric CO_2_, tropospheric ozone pollution, land use change, and increasing nitrogen deposition, etc. on terrestrial carbon storage and fluxes in China and other regions across the globe [15], [29], [30], [31], [32], [33].

In this study, DLEM simulates two kinds of LCLUC: land conversion from natural ecosystem to cropland, and cropland abandonment. In the DLEM model, the balance of soil organic matter depends on the transformation of litter (LIT) to soil organic matter, the fractions of conversion from gross primary production (GPP) to dissolved organic carbon (DOC), the returned organic matter from production decay (PRD) (e.g., manure), the growth of microbe, the methane production from dissolved organic carbon, and the carbon loss from soil organic matter (SOM) decomposition.
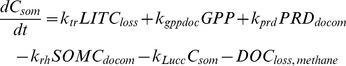
where k*_tr_* is the transfer rate of decomposed LIT to SOM; k*_gppdoc_* is the fraction of GPP converted to soil DOC; k*_prd_* is the returned rate of decomposed (or consumed) PRD to SOM pools as manure; k*_rh_* is the fraction of decomposed SOM that is converted to CO_2_ through heterotrophic respiration; k*_Lucc_* is coefficient for quick carbon loss from SOM due to land use conversion; DOC*_loss,methane_* is DOC consumed for the growth of production of methane. More detailed processes were described in our previous papers [15], [29], [30].

### Input Data Description

The major input data in the DLEM include: (1) daily climatic data (i.e. maximum, minimum, average temperature, precipitation, relative humidity, and radiation) and atmospheric chemistry data (i.e. tropospheric O_3_, atmospheric CO_2_ and nitrogen deposition); (2) soil properties (including soil type, bulk density, depth, pH, soil texture) which are derived from the 1∶1 million soil map based on the Second National Soil Survey of China [34], [35], [36]; (3) contemporary vegetation map for 2000 which was developed from Landsat Enhanced Thematic Mapper (ETM) imagery [37]; (4) long-term land use history which was developed on the basis of three recent (1990, 1995 and 2000) land cover maps and historical census datasets [38], [39]. All the input datasets were developed at the spatial resolution of 10 km×0 km. Detailed information about other input data were described in our previous studies [15], [29], [30], [40].

#### Climate change

**Figure 2:**
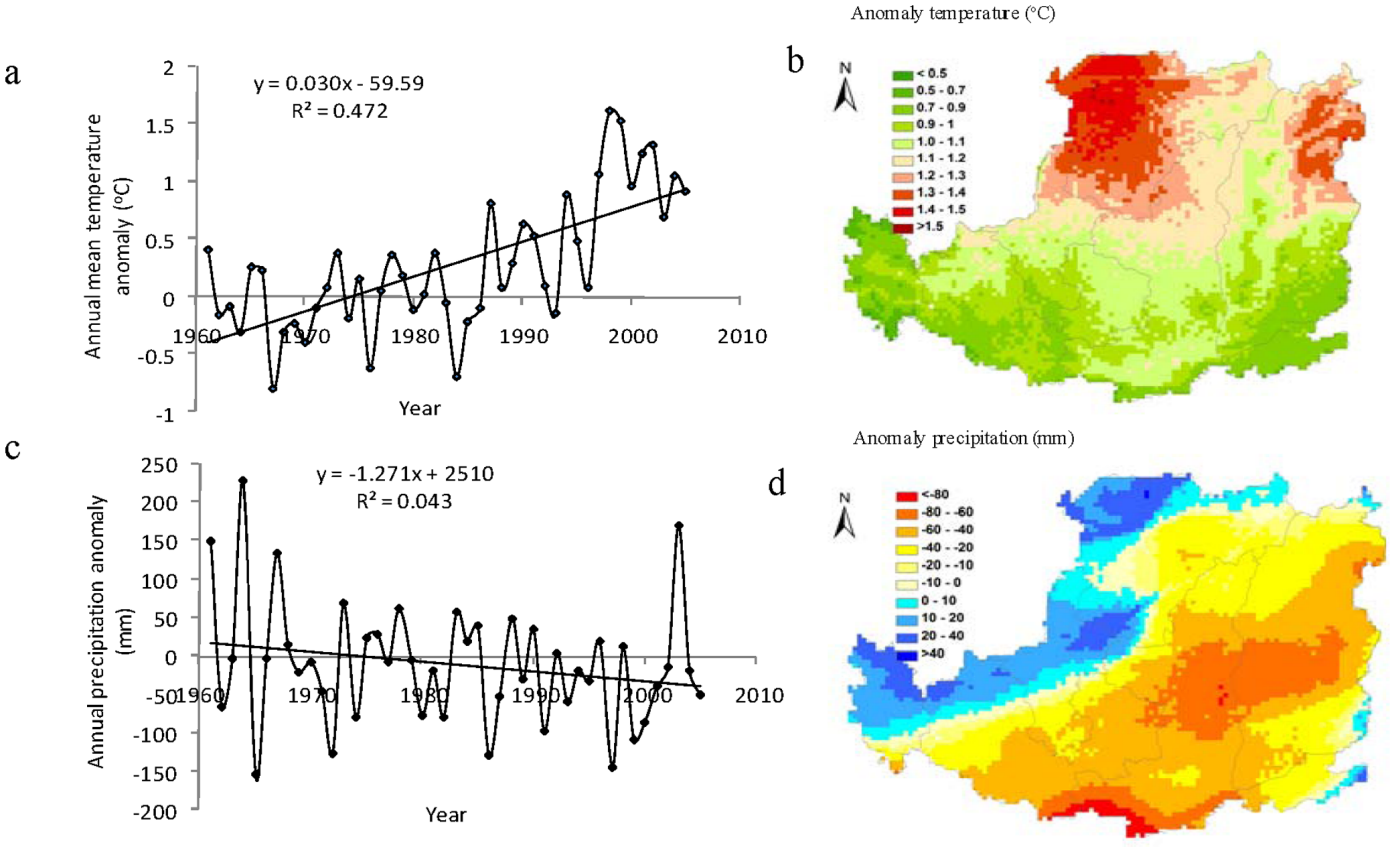
Anomaly of annual mean temperature (a), and precipitation (c) on the Loess Plateau from 1961 to 2005; and spatial distribution of temperature anomaly (b) and precipitation anomaly (d) during 1991–2005 (relative to 1961–1990 average);

Average air temperature on the Loess Plateau increased at a rate of 0.030°C/year from 1961 to 2005 (Figure 2a), higher than those reported for the entire China (about 0.029°C/year) and the global average level (about 0.010°C/year) [41] in the same period. The most rapid increase in temperature occurred during the 1990s. Air temperature increased from the north to the south of the Loess Plateau (Figure 2b), with the greatest increase in the northern Loess Plateau (e.g., the north of Shanxi and the northwest of Inner Mongolia).

The Loess Plateau can be mainly divided into three climate zones according to the precipitation: the northern Loess Plateau with precipitation below 400 mm, the central Loess Plateau with precipitation between 400 and 500 mm, and the southern Loess Plateau with precipitation above 500 mm [42], [43]. The precipitation less than 550 mm/year occurred across most areas of the Loess Plateau. Over the past 45 years, the mean annual precipitation was approximately 423 mm, with the lowest of 288 mm in 1997 and the highest of 661 mm in 1964 (Figure 2c). A slightly decreasing trend at a rate of 1.27 mm/year in precipitation was found on the Loess Plateau from 1961 to 2005. This temporal trend of precipitation was consistent with a previous study based on the meteorological observations according to 99 stations for the period 1956–2005 on the Loess Plateau [44]. Figure 2d further indicated that decreases in precipitation occurred in the most areas of the central and southern Loess Plateau from 1961–1990 to 1991–2005. The regions with the most obvious drying trend were located in the central and southern Loess Plateau, especially in the north of Shaanxi and the center of Shanxi Province with a reduction of more than 80 mm in the recent 15 years (1991–2005) compared to the 1961–1990 average.

#### LCLUC

**Figure 3:**
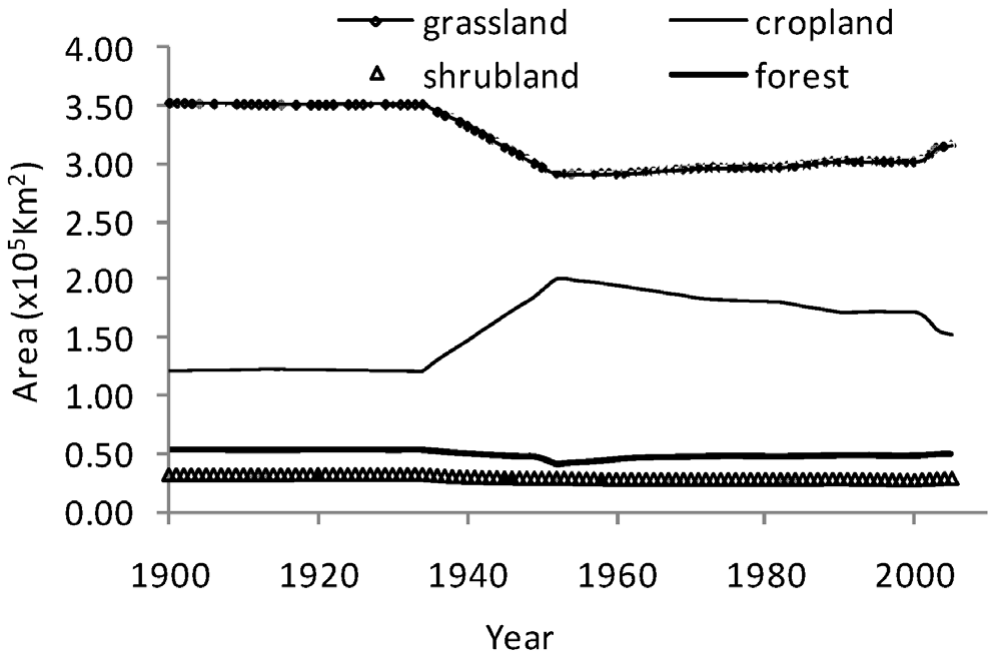
Area of major land use cover on the Loess Plateau during 1900–2005.

**Figure 4:**
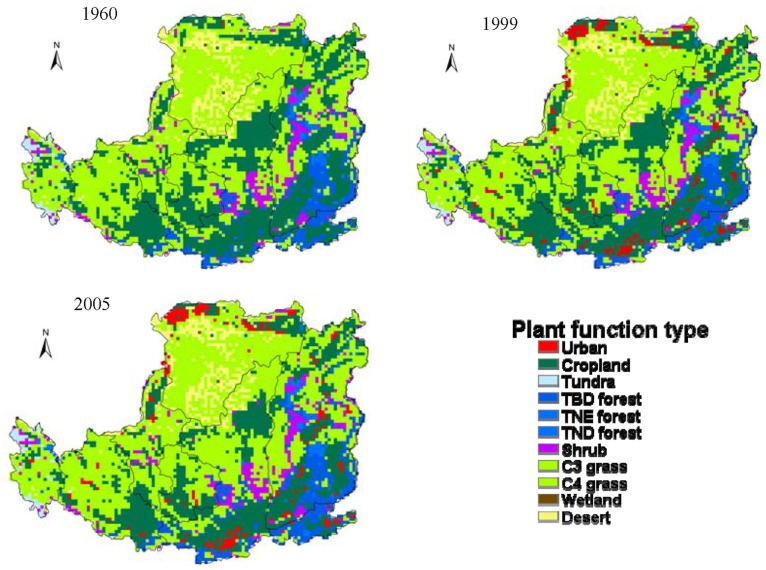
Spatial variations in LCLUC on the Loess Plateau in different year.

Expansion of cropland and pasture was driven by social-economic factors on the Loess Plateau during the past decades, though to some extent, the soil erosion and frequent drought events limited the massive expansion of cropland. Since the 1950s, many measures have been implemented to alleviate and control serious soil erosion on the Loess Plateau. For example, “Grain for Green” project, which was launched in 1999, recommended that cropland with slopes greater than 15^o^ should be converted back to natural vegetation. Driven by such governmental policies, the Loess Plateau experienced a remarkable change in land use, characterizing by a large area of conversion from cropland to grassland/forest. In order to well understand the LCLUC history on the Loess Plateau, we analyzed the spatial and temporal variations from 1961 to 2005 (Figure 3–4). Figure 3 showed that cropland area on the Loess Plateau decreased slowly until 1999, and followed by a sharp decrease. However, the grassland area showed an opposite changing trend in the same periods. Since 1961, the area of cropland decreased by 19.61%, while grassland and forest increased by 7.31% and 6.75%, respectively.

Land use patterns exhibited large spatial variations on the Loess Plateau (Figure 4). Grassland was the dominant vegetation type in the northern Loess Plateau. Cropland, grassland and shrubland were the main vegetation type in the middle Loess Plateau. In contrast, cropland and forest occupied large area of the southern Loess Plateau. Compared to 1960, the coverage of cropland decreased mainly due to land conversion from cropland to grassland, especially in the middle and southern part of Loess Plateau. During 1999–2005, cropland largely shrank in some areas of the middle Loess Plateau, especially in the northern Shaanxi and middle of Shanxi Province. These results were consistent with previous studies [18], [45].

### Model Parameterization and Evaluation

**Figure 5:**
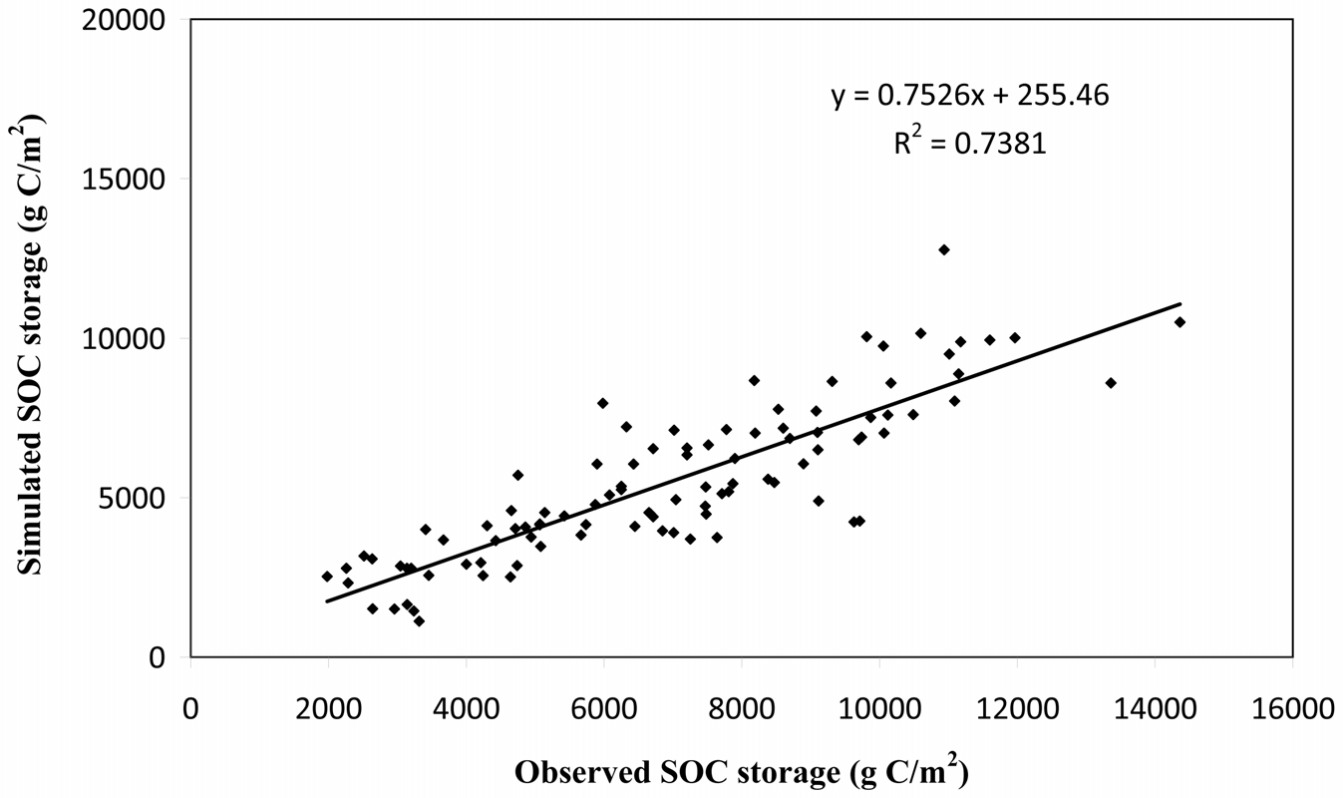
Correlation between simulated and observed SOC storage based on 91 soil samples.

DLEM has been well calibrated and intensively validated against the site-level observed carbon fluxes and pool sizes from Chinese Ecosystem Research Network (CERN) and other previous studies [29], [38], [46]. In this study, we further compared our simulated results with field observation data and survey data from the Second National Soil Survey (1979–1983) to evaluate the model performance in simulating the SOC storage on the Loess Plateau. The simulated SOC storage as influenced by multiple environmental factors significantly correlated with the observed data (R^2^ = 0.738, *p*<0.01, Figure 5), indicating that DLEM could capture the spatial and temporal patterns of SOC storage on the Loess Plateau. Several factors may contribute to the difference between model results and field observations. First, the input datasets were developed at a spatial resolution of 10 km×10 km for driving DLEM simulations. Each grid was assumed to have the uniform climate, land use type, and vegetation cover in the model. As a well-known climate-sensitive zone and a fragile ecological belt, some subtle changes in climate, land use or other factors on the Loess Plateau might cause large difference in the SOC storage even within one grid cell. This difference in the same grid will be reflected in the field experiments but neglected in the model simulations. Second, the shortage of field observation data may weaken the capability of model to realistically capture the magnitude and patterns in SOC changes, which has long been identified as one of the biases in the large-scale model development. In addition, model simplification and neglecting microbial biomass might be another potential reason [13], [30].

### Simulation Experimental Design and Model Run

In this study, four main simulation experiments were designed to analyze the effects of climate change alone, LCLUC alone, the interaction of climate and LCLUC, and the combined effects of all environmental factors on the SOC storage on the Loess Plateau (Table 1). The experiment I was designed to provide a ‘best-estimate’ of spatial and temporal patterns of SOC driven by major environmental factors changes including climate variability, LCLUC, elevated CO_2_, N fertilizer, N deposition, and O_3_ pollution, etc. In experiment II and III, we simulated the contributions of climate variability alone and the LCLUC alone, respectively. In the experiment IV, we tried to understand the interactive effect between climate change and LCLUC on the SOC storage. The model simulations began with an equilibrium run to obtain the baseline carbon pools for each grid. A spin-up of about 100 years was applied if the climate change was included in the simulation experiments. Finally, the model was run in transient mode driven by the daily climate data and other time-variant or invariant input data.

**Table 1:** Simulation experiments.

Simulation Experiment	Environmental factors		
	Climate	LCLUC	Others
I. All	1960–2005	1960–2005	1960–2005
II. Climate only	1960–2005	1960	1960
III. LCLUC only	1960	1960–2005	1960
IV. Climate-LCLUC	1960–2005	1960–2005	1960

Notes: Climate-LCLUC means the combination effects of climate and LCLUC. Others include the effects of atmospheric CO_2_, ozone pollution (AOT 40 index), nitrogen deposition, nitrogen fertilizer application, etc. All means the simulation experiment which includes all above environmental factors.

## Results and Analysis

### Temporal Changes in SOC on the Loess Plateau

**Figure 6:**
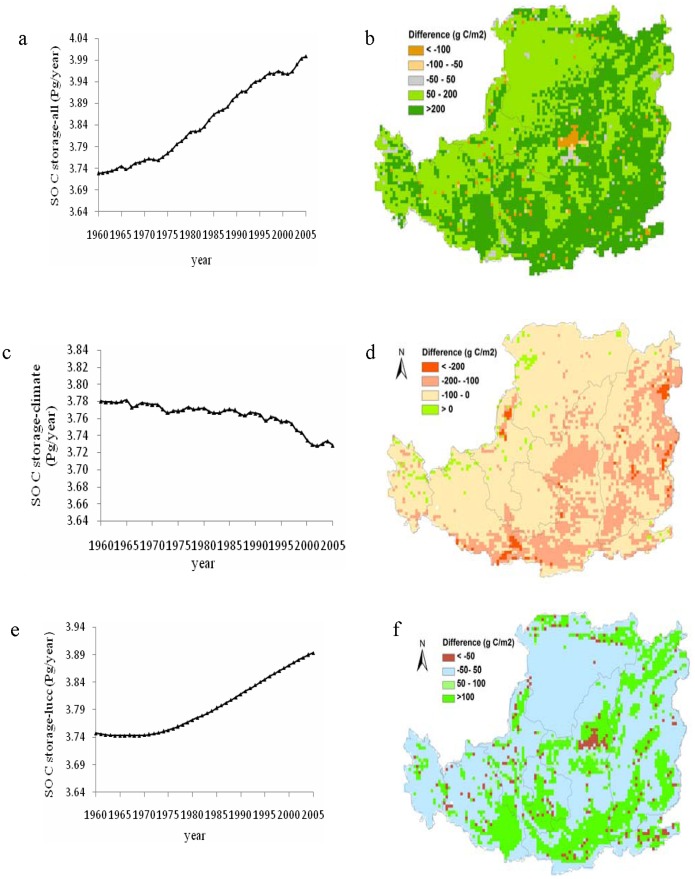
Spatiotemporal variations of SOC storage under experiments I, II, and III on the Loess Plateau from 1960–2005. Note: The "difference" means change in SOC storage between 2005 and 1960.

Model simulation indicated that the SOC storage over the entire Loess Plateau displayed substantial temporal and spatial variations in the context of multiple environmental factors changes (Figure 6a, b). As a whole, the combination of all these environmental factors considered in this study (e.g. climate, LCLUC and others environment factors) caused a net increase of about 0.27 Pg C in SOC storage from 1961 to 2005. The SOC storage kept relatively stable before the 1970s, and then gradually increased in the following two decades, and rapidly increased since 2000 (Figure 6a). Our further analysis found that the decrease of cropland area was relatively slower from the 1960s to 1999, and became rapid after then. Most of the abandoned cropland were replaced by grassland (Figure 3). Our results implied that temporal pattern in SOC change was partly related to the land use change on the Loess Plateau.

### Spatial Variation in SOC on the Loess Plateau

We found that the spatial patterns of SOC storage were primarily controlled by the precipitation distribution. Spatially, the SOC storage increased gradually from the north to the south along an increasing precipitation gradient on the Loess Plateau. Large SOC increases were found in the southern and central Loess Plateau (south of Shaanxi and Shanxi Province in particular); and some other areas show a slight increase of SOC storage in the past decades. Due to changes in multiple environmental factors, the SOC storage increased throughout the majority of the Loess Plateau over the past 45 years (Figure 6b). Some areas showed a significant increase in SOC storage of more than 200 g C/m^2^. However, a significant decrease occurred in the middle of central Loess Plateau (especially in the Northern Shaanxi Province), where experienced the most obvious warming and drying tendency in the past decades (Figure 2 b,d), releasing more than 100 g C/m^2^ during the study period. We further found that the SOC increase in the northern Loess Plateau was lower than that in the southern Loess Plateau during the past decades.

### Individual Factorial Contributions to Changes in SOC Storage

To well understand the influence of climate on the SOC storage, we simulated the SOC storage on the Loess Plateau under the influence of climate change alone. DLEM simulated results showed that SOC storage decreased by about 0.05 Pg C with substantial inter-annual fluctuations throughout the Loess Plateau from 1961 to 2005 (Figure 6c). SOC storage showed a slowly decreasing trend before the late 1990s, followed by a sharp decrease until the early 21^st^ century. Since 1961, SOC storage decreased in most areas of the Loess Plateau under the influences of the climate, with a maximum carbon release of 200 g C/m^2^ in some areas of the southern and the central Loess Plateau (Figure 6d). Figure 6d also indicated that SOC storage decreased in the southeastern humid monsoon climatic regions, and to a lesser extent in the continental dry climatic regions in the northern Loess Plateau during 1961–2005.

Considering the single effect of LCLUC, our results showed that the SOC storage increased by 0.14 Pg C during 1961–2005 (Figure 6e). We found that the most rapid increase in the SOC storage occurred after the late 1970s (Figure 6e). DLEM simulation results also showed that obvious increases of SOC storage were found in most areas of the central and the southern Loess Plateau (Figure 6f).

### Relative Contributions of Climate, LCLUC, and their Interactions

**Figure 7:**
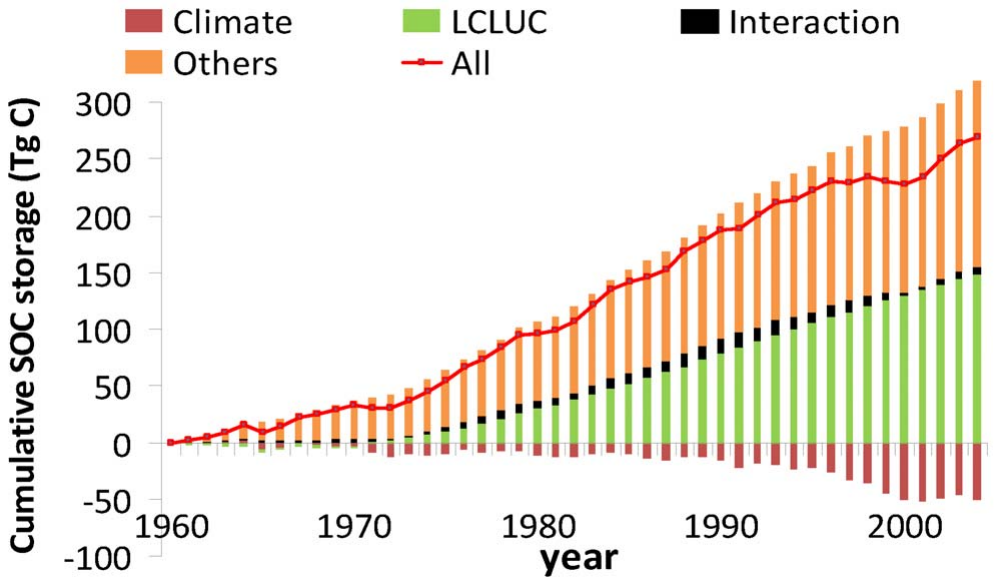
Contributions of multi-factor global changes to accumulative SOC change during 1961–2005. Note: “interaction” referrers to the interactive effects between climate and LCLUC.

Our simulated results indicated that with multiple environmental changes, SOC continuously increased from 1961 to 2005 on the Loess Plateau, resulting in a net increase in SOC storage by 0.27 Pg C (5.97 Tg C/year) (Figure 7). Among these factors, LCLUC was obviously the major factor affecting the magnitude of SOC change on the Loess Plateau, leading to a significant increase (0.14 Pg C) in SOC storage, accounting for 55% of the net increase in SOC in the past 45 years. However, in the same period, warming and drying climate greatly reduced SOC storage by 0.05 Pg C, approximately 19% of the total SOC storage change. The interaction between climate and LCLUC contributed to the net SOC increases by 3%.

## Discussion

### SOC Storage Change on the Loess Plateau

In this study, the combination of all environmental factors (climate, LCLUC and others environment factors) caused a net increase of about 0.27 Pg C in SOC storage, indicating that soil acted as a weak carbon sink on the Loess Plateau during the past 45 year. The temporal pattern of SOC storage was largely influenced by land use change and relevant land use policies.

Except the central Loess Plateau, the SOC storage increased throughout the entire region, with a smaller sink in the northern Loess Plateau and a larger sink in the southern Loess Plateau during 1961–2005, which is consistent with previous reports [43], [47], [48]. The less increase of SOC storage in the northern Loess Plateau might be due to more sandy soil, lower soil carbon input from plant biomass, and larger soil carbon loss from erosion. This is consistent with previous reports, indicating the soil in the northern Loess Plateau contained more sand which could accumulate less carbon than the soil with more clay [49], [50], [51], [52]. On the other hand, the area of forest and shrubland, characterized by higher aboveground biomass and productivity than cropland and grassland [43], [53], decreased from the south to north over the Loess Plateau (Figure 4). This also contributed to realtively less SOC increase in the northern Loess Plateau. In addition, the extensive soil erosions in the northern Loess Plateau was suggested to further explain the lower contents of SOC storage [2], [17], [54].

### Comparisons with Field Observations

Our estimation of SOC storage and its change on the Loess Plateau were comparable to other studies. Based on the 0–100 cm SOC data collected from 382 sampling sites across the entire Loess Plateau in 2008, Liu et al. [55] indicated that mean SOC storage was 7.70 kg C/m^2^. Using data gathered by the Second National Soil Survey of China (1979–1983), Xu et al. [56] estimated that SOC storage was 1.07 Pg in 0–20 cm soils layers on the Loess Plateau region (with a land area of 429,800 km^2^). A soil survey conducted throughout the Loess Plateau region indicated that the SOC storage amounted to 1.23 Pg C in the 0–20 cm soil layers with land area of 592,900 km^2^ during 1985–1988, [55]. Converting SOC storage from different soil depth to the same soil depth according to the summary of the vertical distribution of soil organic in the 0–100 cm soil [31], SOC storage were 6.22 kg C/m^2^ and 5.19 kg C/m^2^ in Xu et al.’s [57] and Liu et al.’s [55] reports (be mentioned but not published) respectively. In the same soil depth, DLEM-estimated average SOC storage was 6.45 kg C/m^2^ in 2005, which falls in the range of previous studies.

Tian et al. [7] estimated an average SOC sink of 94 Tg C/year in the terrestrial ecosystem of China as influenced by multiple global change factors during 1961–2005. Our results showed that soil on the Loess Plateau acted as a weak sink of 5.97 Tg C/year under the combined experiment during the same period. Considering that the Loess Plateau accounts for approximately 6.5% of the country’s land surface, implying that the SOC storage on the Loess Plateau was close to the country-level average. At national level, Wang et al. [35] suggested that SOC storage in China decreased by about 5.69 g C/m^2^ per year during the 1960s–1980s, while our study showed that the SOC storage on the Loess Plateau significantly increased by 4.30 g C/m^2^ per year during the same period. This indicated that successful implementation of land conservation measures was beneficial and effective to reduce soil erosion and improve soil properties, thus enhance the soil carbon sequestration over the Loess Plateau.

### Climate Controls on the Spatiaotemporal Patterns of SOC Storage

Climatic factors, especially precipitation and temperature, play an important role in long-term variations of SOC due to their effects on the quantity and quality of organic residue inputs and on the rates of soil organic matter and litter decomposition [1], [12], [58]. DLEM-simulated results showed that SOC storage decreased by about 0.05 Pg C with a significant inter-annual fluctuations throughout the Loess Plateau from 1961 to 2005 as influenced by climate change alone (Figure 6c). As shown in Figure 2, precipitation decreased at a rate of 1.27 (mm/year) during 1961–2005, which was a notable factor that influenced the change of SOC on the Loess Plateau, especially after 1990. Meanwhile, increased air temperature (0.030°C/year) might accelerate the evapotranspiration and potentially aggravate the water deficiency, thus cause the formation of drying soil layer and suppress the growth of vegetation. Due to the warming and drying climate, dried soil layer was widely distributed in the hilly and gully areas of the Loess Plateau. The development of drying soil layer has been regarded as a key cause for the decrease of SOC storage in some areas on the Loess Plateau in the past decades [59].

Climate also had a substantial effect on the spatial distribution of SOC storage. Our results suggested that SOC storage decreased in most areas of the Loess Plateau under the influences of the climate change since 1961 (Figure 6d). This result could be explained partly by the distribution of temperature and precipitation, which is well-known to have a positive relationship with SOC decomposition [60]. During 1961–2005, both temperature and precipitation were higher in the central and southern Loess Plateau, compared to the northern Loess Plateau. Therefore, the higher loss of soil carbon from decomposition might be an important cause for lower SOC accumulation in the central and southern Loess Plateau. This result was supported by previous studies [43], [50], [57].

### Land Use Controls on the Spatiaotemporal Patterns of SOC Storage

During the past decades, the Loess Plateau has experienced a complex change in land use pattern (Figure 3). Considering the LCLUC alone, DLEM-simulated results showed that the SOC storage increased by 0.14 Pg C during 1961–2005 (Figure 6e), which was significantly larger than the influence of climate change alone. However, in the first few years, the SOC changed slowly, even had a decreasing tendency in the 1960s, and then increased gradually since the 1980s.

Since the 1950s, various soil and water conservation measures including afforestation, cropland abandonment, and terrace construction etc., have been implemented in this region [17], [18]. All of these measures directly or indirectly affected the vegetation cover and further influenced the SOC storage on the Loess Plateau. Previous studies demonstrated that soil can lose up to 20–40 percent of organic carbon into the atmosphere when perennial vegetation land was converted into cultivation land [61], [62]. On the contrary, conversion from cropland to perennial vegetation land or shrubland was found to accumulate SOC by increasing carbon derived from new vegetation and decreasing carbon loss from decomposition and erosion [11], [43], [51], [63]. Liu et al. [47] reported that SOC in shrubland, which was converted from cropland in 1985, were 27.7%–34.8% higher than that of the cropland in 2010 on the Loess Plateau. Fu et al. [64] suggested that cropland abandonment significantly increased the density and stock of SOC in 0–100 cm soil profiles on the Loess Plateau. Feng et al. [48] found a total of 96.1 Tg of additional carbon had been sequestered on the Loess Plateau since China’s “Grain for Green” program during 2000–2008, by using remote sensing techniques and ecosystem modelling. Our results are comparable with those previous findings.

LCLUC might induce an immediate change in vegetation coverage but a lagged effect on the change of SOC storage [65]. In this study, the SOC changed slowly, and even had a decreasing trend in the 1960s. We also found that the most rapid increase in the SOC storage occurred after the late 1970s, which might be partly due to extensive and conducted tree planting projects for alleviating soil erosion. The large-scale cropland abandonment in response to the recently implemented “Grain for Green” policy also contributed to the increase of SOC storage on the Loess Plateau. The plantation from “Grain for Green” Project would keep a large proportion of carbon in wood which need long time to return to soil. During the initial period of land conversion from cropland to forests, leaf biomass of trees were very low, so the litters on the ground decreased and resulted in a slight change (even decrease) in SOC. During the period of implementing “Grain for Green” project, litters on the ground accumulated gradually with the growth of planted trees, resulting in increasing SOC storage after a certain time period. Compared with simulated results in the context of multiple environmental factors, a similar temporal pattern for the SOC change was found when considering LCLUC alone from 1961 to 2005 (Figure 6a,e). This further implied that LCLUC was the dominant factor controlling temporal variations of SOC storage.

DLEM simulated results also showed obvious increases of SOC storage in most areas of the central and southern Loess Plateau (Figure 6f). Further analysis found that changes in SOC storage were smaller in the LCLUC alone simulation experiment than that with the combination of all environmental change (Figure 6b). However, SOC storage changed slightly in most areas of the northern Loess Plateau from 1961 to 2005, which were also suggested by other research results (e.g., [18], [43]).

### Interactive Effects of LCLUC and Climate on SOC Storage

Based on DLEM simulations, Tian et al. [7] found that LCLUC accounted for 17% of the net carbon increase, but climate change reduced it by 4% in China in the past decades. However, we found that LCLUC increased SOC storage by 0.14 Pg C, accounting for 55% of the net increase in SOC in the past 45 years over the Loess Plateau. In the same period, warming and drying climate greatly reduced SOC storage by 0.05 Pg C, approximately 19% of the total SOC storage change. These results implied that the SOC was more sensitive to LCLUC and climate change on the Loess Plateau comparing to other regions in China. In the past decades, LCLUC, particularly in the southern Loess Plateau, enhanced the vegetation coverage and reduced the anthropogenic disturbance, which further enhanced the SOC storage.

The interaction between climate and LCLUC contributed to the net SOC increases by 3%. Our results also showed that the interactive effects among environmental factors can’t be neglected in attributing the changes of SOC storage in response to environmental factors on the Loess Plateau. Although the interaction among environmental factors has been recognized long before [66], most of the field experiments still overlook it. This study further demonstrated that the modeling approach may serve as one complementary tool for the field experiments in addressing interactive effects among multiple environmental factors.

### Effects of Cropland Abandonment on SOC Storage

Cropland abandonment and natural vegetation recovery were important implementations to mitigate soil loss on the Loess Plateau. Previous studies demonstrated that former land use types, soil property, climate change, and soil management practices were crucial to changes of SOC storage in the establishment of perennial vegetation type [49], [50]. Other factors, such as the abandonment age, have also been found to play a significant role in SOC accumulation and should not be ignored [4], [50].

**Figure 8:**
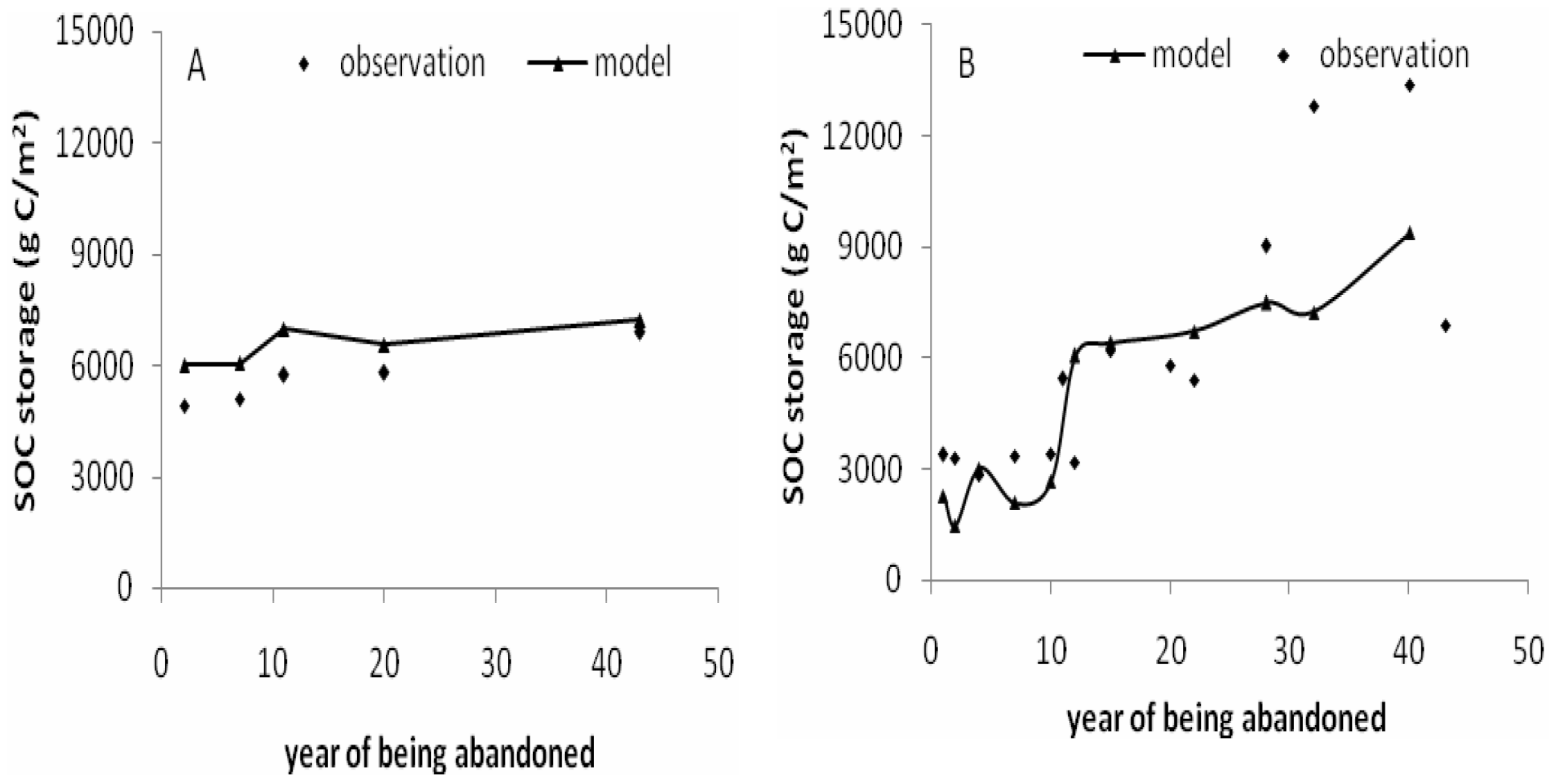
Comparison between simulated (DLEM) and observed SOC storage. Notes: A: cropland abandoned inYuzhong (Gansu Province), the observation data come from Jinping Jiang et al. [67]; B: cropland abandoned in Yan’an (Shaanxi Province), the observation data come from Junmin Wang et al. [2].

In this study, we chose two sites (Yuzhong City and Yan’an City) (Marked in Figure 1) located on the Loess Plateau to explore SOC storage pattern at different abandonment stages (or restoration age) and to further evaluate the DLEM simulated results against the observations (Figure 8). It showed that DLEM results could well capture the distribution characteristics of the SOC storage in different abandonment stages. Both model results and field observations demonstrated that the restoration age played a key role in SOC accumulation. Jiang et al. [67] indicated that SOC storage changed little in early abandonment stage in a semiarid hilly area of Yuzhong City, followed by an obvious increase after 9–12 years, and then increased stably (Figure 8A). Wang et al. [2] found the similar temporal pattern through studying the change of SOC storage during different successional stages of rehabilitated grassland in Yan’an City. Compared to cropland, the rehabilitated grassland had a lower SOC storage at the early stage (1–12 years). However, SOC storage was higher than that in cropland after 15 years and then increased steadily (Figure 8B). Generally, long-term abandonment (>10 years) and the following colonization of natural vegetation could lead to substantial increase of SOC storage. The difference of the SOC storage between two sites over the early abandonment stage might be attributed to difference in environmental factors such as climate and soil property. Yan’an City is located in a semi-arid and warm temperate zone with less precipitation and more obvious drying and warming tendency, compared with Yuzhong City during the past years. This also partly explained the lower SOC storage in cropland in this climate zone than others over the Loess Plateau.

Our results indicated that cropland abandonment could increase SOC storage, improve soil quality and promote ecosystem restoration, especially in the warm and dry climate zone. However, these effects will emerge after a relative longer time period (e.g., >10 years) under local environmental conditions.

### Uncertainties

This study examined temporal and spatial patterns of SOC storage and attributed these patterns to multiple environmental factors on the Loess Plateau during 1961–2005. There were several uncertainties which need to be addressed in our future work. First, impacts of some ecological processes, such as soil erosion, were not separated from other environmental factors in this study. The soil erosion area could be as high as 45.4×10^4^ km^2^ (72.3% of the land area) over the Loess Plateau. Soil erosion has long been identified as one of key factors controlling the change of SOC storage [47]. Further efforts should be put on interactions among soil erosion and other environmental factors. Second, the input datasets were developed at the spatial resolution of 10 km×10 km which was the finest dataset for the Loess Plateau. However, it is still difficult to capture subtle change of SOC storage due to high heterogeneity in land surface processes. In the long run, finer gidded datasets would be greatly helpful for further quantifying temporal and spatial changes in SOC storage on the Loess Plateau. In addition, the uncertainties from other input data, model structure, and parameterization need to be further specified in the furture efforts.

## Conclusions

This study examined effects of climate and land use changes on SOC storage on the Loess Plateau of China in the context of multiple global changes by using an integrated ecosystem model. The results showed that temperature on the Loess Plateau has significantly increased, while precipitation slightly decreased during 1961–2005. Meanwhile, this region experienced a remarkable change in land cover and land use, characterized by conversions from cropland to grassland/forest owing to the government policies to alleviate soil and water losses during the past decades. The overall change in SOC storage due to multiple environmental factors was estimated to be a net increase of SOC storage by 0.27 Pg C during 1961–2005, indicating that soil on the Loess Plateau acted as a carbon sink in this period. Among multiple factors, LCLUC led to a significant increase in SOC storage of 0.14 Pg C, accounting for 55% of the net increase in SOC. In contrast, climate change reduced SOC storage by 0.05 Pg C (approximately 19% of the total SOC change). The interaction of climate and LCLUC accounted for 3% of the net increase in SOC. Our results were consistent with field observation data and both of them suggested that SOC storage could be enhanced significantly by the conversion of cropland to grassland along with the increasing abandonment age on the Loess Plateau. However, the magnitude could be influenced by local environmental conditions.

This study provides the first attempt to quantify relative effects of multiple environmental factors (climate and LCLUC in particular) on regional SOC storage on the Loess Plateau over the past decades. The results drawn from this study provide insight for land management as well as policy-making to enhance carbon sequestration and alleviate the serious soil erosion conditions on the Loess Plateau. To reduce uncertainties in estimating effects of climate and land use changes on SOC storage, it is needed to put further efforts in developing more reliable and fine-resolution input data and improve model representation of some other processes relevant to SOC, such as soil erosion.
